# The association between obesity index and hypertension in type 2 diabetes: a cross-sectional study

**DOI:** 10.3389/fendo.2026.1998153

**Published:** 2026-10-27

**Authors:** Yuwei Xing, Yishan Yin, Qianqian Zhao, Lili Zhang, Xian Xiu

**Affiliations:** 1 Department of Endocrinology, The Second Hospital of Shijiazhuang, Shijiazhuang, China; 2 Department of Orthopedics, The Armed Police Forces Hospital of Shandong, Jinan, China

**Keywords:** Chinese visceral adiposity index, hypertension, lipid accumulation product, restricted cubic spline, sex-specific analysis, type 2 diabetes mellitus

## Abstract

**Background:**

The present study explored the association between the Chinese visceral adiposity index (CVAI) and lipid accumulation product (LAP) and hypertension in type 2 diabetes mellitus (T2DM) subjects.

**Methods:**

A total of 4,155 T2DM individuals were enrolled from the Second Hospital of Shijiazhuang in this cross-sectional investigation. We employed multivariable logistic regression, restricted cubic spline (RCS) modeling, threshold effect assessment, and stratified subgroup analyses. Missing covariates were addressed through multiple imputation, while complete-case data were used for sensitivity evaluation.

**Results:**

Among 4,155 participants (68.5% hypertensive), CVAI and LAP were positively associated with hypertension (P for trend < 0.001). Q4 vs. Q1 OR was 3.27 (95% CI: 2.66–4.02) for CVAI and 3.01 (95% CI: 2.43–3.72) for LAP. RCS evaluation demonstrated a significant nonlinear relationship between LAP and hypertension (P for non-linearity < 0.001), identifying a threshold value of 44.187 (95% CI: 42.141–46.233). CVAI showed linear associations in both sexes (P for non-linearity: 0.177 males, 0.055 females). Subgroup analyses showed consistent associations across strata (all P for interaction > 0.05).

**Conclusions:**

CVAI and LAP are associated with hypertension in T2DM patients. LAP exhibits a nonlinear relationship with a threshold effect at approximately 44.2.

## 1 Introduction

Type 2 diabetes mellitus (T2DM) affects over 537 million adults globally, with projections reaching 783 million by 2045 (1). In China, prevalence reached 12.4% in 2018, affecting approximately 130 million individuals (2). Given that T2DM commonly advances to cardiovascular and renal complications (3), timely risk assessment is essential. Obesity is a well-established modifiable contributor to elevated blood pressure (4); however, conventional measures such as body mass index (BMI) and waist circumference (WC) fail to capture visceral fat distribution adequately (5, 6). Metrics integrating metabolic and abdominal fat domains—specifically the Chinese visceral adiposity index (CVAI) and lipid accumulation product (LAP)—have been advocated for evaluating cardiometabolic risk (7, 8), yet their discriminative accuracy among T2DM individuals undergoing profound metabolic changes remains uncertain (7).

Sex and age critically modify the adiposity–hypertension relationship. Menopausal transitions diminish the diagnostic specificity of conventional adiposity metrics (9), and a nationwide study confirmed sex-specific CVAI–hypertension associations, with a significantly stronger effect in women (P for interaction < 0.001) (8, 10). To address these shortcomings, we employed two complementary indices: CVAI, validated against CT-measured visceral fat in Chinese adults and integrating age, BMI, WC, triglycerides, and HDL-C (9); and LAP, constructed from WC and fasting triglycerides, reflecting systemic lipid overaccumulation (11). Comparing these two complementary indices within the same cohort allowed us to determine whether visceral adiposity or peripheral lipid overaccumulation is more closely related to hypertension in patients with type 2 diabetes. The 2021 Lancet Diabetes & Endocrinology series recognized that adiposity-distribution indices outperform BMI in capturing cardiometabolic risk in Chinese populations (12, 13).

CVAI has been associated with incident hypertension in prospective cohorts (14), demonstrates superior discriminative value among adiposity indices (15), and predicts cardiovascular events in T2DM (7). LAP captures abdominal lipid deposition with robust links to elevated blood pressure and mortality (7). Nonetheless, debates persist regarding the dose–response shape of these associations (8, 10, 16), their consistency across cohorts (4, 11, 17), and whether CVAI or LAP exhibits a stronger correlation with hypertension in T2DM (7, 18). Gender-specific evidence remains limited (10, 15), and direct comparative evaluations between these measures are scarce. This study therefore aimed to characterize the dose–response relationships of CVAI and LAP with hypertension in T2DM patients, incorporating sex-stratified analyses.

CVAI and LAP capture distinct but complementary physiological domains. CVAI is a sex-specific composite of age, BMI, waist circumference, triglycerides, and HDL-cholesterol, originally developed and validated against computed tomography–measured visceral fat area in Chinese adults, and therefore serves primarily as a surrogate of visceral adiposity. LAP, in contrast, is a simple product of waist circumference and fasting triglycerides that reflects systemic lipid overaccumulation and cardiometabolic toxicity. Because the two indices differ in composition—CVAI additionally incorporates age and HDL-cholesterol and is calibrated to visceral fat, whereas LAP emphasizes the waist–triglyceride interaction—comparing them within the same data allows us to determine whether visceral adiposity or peripheral lipid overaccumulation is more closely related to hypertension in patients with type 2 diabetes.

Accordingly, this cross-sectional study was conducted to examine the strength of the associations of CVAI and LAP with hypertension in patients with T2DM, and to characterize their dose–response relationships using restricted cubic spline and threshold effect analyses. Additionally, we explored whether gender serves as an effect modifier on these connections, seeking to deliver a gender-specific profile of such linkages and to formulate propositions for subsequent prospective investigations.

## 2 Materials and methods

### 2.1 Study design and population

This cross-sectional study was carried out within the Endocrinology Division of the Second Hospital of Shijiazhuang, leveraging information extracted from the institutional electronic medical record (EMR) platform. The study period spanned from January 2024 to December 2025. Ethical clearance was obtained from the Institutional Review Board of the Second Hospital of Shijiazhuang City (Approval No.: SEY202011117). Given its retrospective design and the complete de-identification of all participant records, the review board exempted the need for written informed consent. All procedures adhered to the principles outlined in the Declaration of Helsinki and applicable institutional protocols.

Inclusion required participants to be ≥18 years of age with a verified diagnosis of type 2 diabetes mellitus (T2DM) established according to American Diabetes Association (ADA) or World Health Organization (WHO) criteria (19) (1): typical diabetic manifestations together with a random venous plasma glucose ≥11.1 mmol/L (2); a fasting plasma glucose ≥7.0 mmol/L; or (3) a 2-hour post-load glucose ≥11.1 mmol/L during a 75-g oral glucose tolerance test (OGTT). For individuals without symptoms, confirmatory laboratory testing on a subsequent occasion was obligatory. Participants were also required to have key anthropometric measurements (height, weight, waist circumference) and hypertension information available for calculation of the required obesity indices.

Individuals were disqualified for the following reasons: presence of type 1 diabetes, gestational diabetes, or miscellaneous and secondary diabetes variants; absence of essential data needed for computing adiposity indices or establishing hypertensive status; subjects whose arterial pressure or somatometric readings were rendered invalid by acute critical illness, urgent medical circumstances, or suboptimal measurement environments; advanced renal failure requiring chronic renal replacement therapy; or medically confirmed secondary hypertensive disease.

The initial screening encompassed 6,361 individuals. After sequential exclusion of non-T2DM patients (n = 688), patients with missing hypertension data (n = 3), and patients with missing lipid accumulation product (LAP) or Chinese visceral adiposity index (CVAI) data (n = 1,515), the final analytical cohort comprised 4,155 patients with T2DM, including 2,846 with hypertension and 1,309 without hypertension. A flow diagram of participant selection is presented in **Figure 1**.

**Figure 1:**
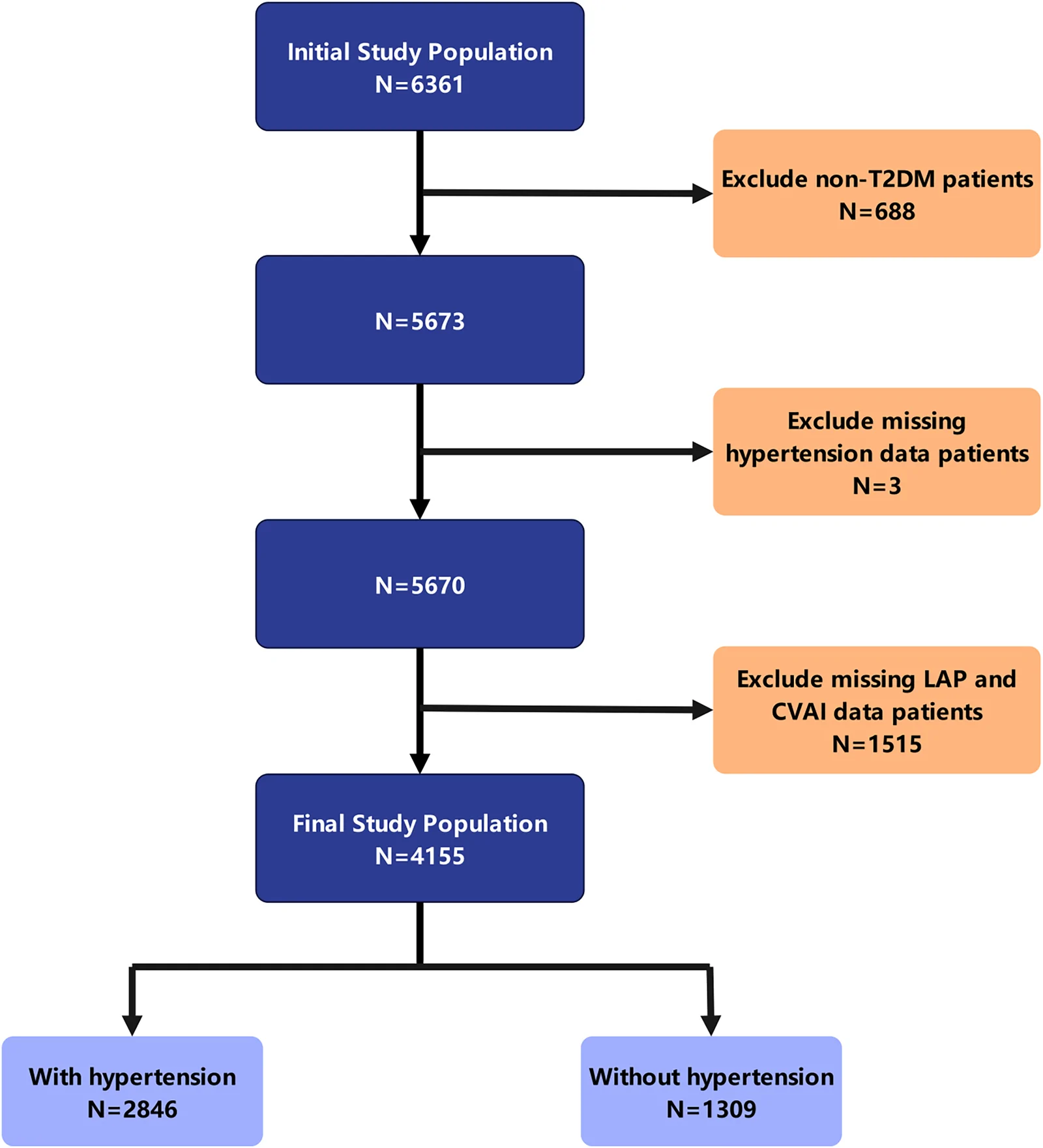
Flowchart of participant selection. The flowchart illustrates the process of selecting the final study cohort from the initial population. A total of 6,361 patients were initially screened. After sequential exclusion of non-type 2 diabetes mellitus (non-T2DM) patients (*n* = 688), patients with missing hypertension data (*n* = 3), and patients with missing lipid accumulation product (LAP) or Chinese visceral adiposity index (CVAI) data (*n* = 1,515), the final analytical cohort comprised 4,155 patients with T2DM, including 2,846 with hypertension and 1,309 without hypertension. CVAI, Chinese visceral adiposity index; LAP, lipid accumulation product; T2DM, type 2 diabetes mellitus.

### 2.2 Measurements

#### 2.2.1 Anthropometric measurements

Subjects were weighed and measured in light indoor attire without footwear. An electronically calibrated column-type apparatus yielded weight readings precise to 0.1 kg. Stature was measured using a wall-fixed stadiometer, accurate to 0.1 cm. Body mass index (BMI) was computed as weight (kg) divided by the square of height (m) (kg/m²). Waist circumference (WC) was measured at the midpoint between the lower costal margin and the iliac crest at the end of normal expiration, to the nearest 0.1 cm. All measurements were performed twice, and the average was recorded.

#### 2.2.2 Blood pressure assessment and hypertension definition

Blood pressure was measured using a standardized protocol. Hypertension was defined based on the electronic medical record (EMR) system: participants with a recorded diagnosis of hypertension or current use of antihypertensive medication were classified as hypertensive.

#### 2.2.3 Laboratory assays

Peripheral venous samples were drawn from every subject following a minimum 8-hour overnight abstinence from food. Evaluated laboratory markers comprised glycosylated hemoglobin (HbA1c), fasting plasma glucose, estimated glomerular filtration rate (eGFR, computed using the CKD-EPI equation), triglyceride levels, total cholesterol, high-density lipoprotein cholesterol (HDL-C), and low-density lipoprotein cholesterol (LDL-C). All analyses were performed using standardized laboratory protocols.

#### 2.2.4 Obesity index calculations

The following obesity indices were calculated using established formulas (20):

1. Chinese Visceral Adiposity Index


$$\text{Males:CVAI}=-267.93\;+\;0.68\;\times \text{ age }\left(\text{year}\right)+0.03\;\times \text{ BMI }\left(\text{kg}/{\text{m}}^{2}\right)+4.00\times \text{WC}\left(\text{cm}\right)+22.00\times \text{lg }\left(\text{TG}\right)\left(\text{mmol}/\text{L}\right)-16.32\times \text{HDL}-\text{C }\left(\text{mmol}/\text{L}\right);$$

$$\text{Females}:\text{CVAI}=-187.32\;+\;1.71\times \text{age}\left(\text{year}\right)+4.23\times \text{BMI}\left(\text{kg}/{\text{m}}^{2}\right)+1.12\times \text{WC}\left(\text{cm}\right)+39.76\times \text{lg}\left(\text{TG}\right)\left(\text{mmol}/\text{L}\right)-11.66\times \text{HDL}-\text{C}\left(\text{mmol}/\text{L}\right);$$

2. Lipid accumulation product

- Males: LAP = TG (mmol/L) × [WC (cm) − 65]
- Females: LAP = TG (mmol/L) × [WC (cm) − 58]

Every parameter was computed separately on two occasions by distinct investigators. Any divergent results were adjudicated by an additional experienced investigator.

#### 2.2.5 Covariate assessment

The following covariates were collected from the EMR system: age, sex, diabetes duration, HbA1c, eGFR, smoking history (binary: yes/no), drinking history (binary: yes/no), and use of antidiabetic medications including metformin, sulfonylureas (SU), α-glucosidase inhibitors, thiazolidinediones (TZD), GLP-1 receptor agonists, DPP-4 inhibitors, SGLT2 inhibitors, insulin, and repaglinide (all binary: use or non-use, based on medical prescription records).

### 2.3 Statistical analyses

Continuous variables conforming approximately to a Gaussian distribution were expressed as mean ± standard deviation (SD), whereas non-normally distributed data were displayed as median with interquartile range (IQR); categorical data were reported as frequencies (percentages). Intergroup comparisons utilized Student’s t-test for normally distributed continuous data, the Mann–Whitney U test for nonparametric continuous data, and either the chi-square test or Fisher’s exact test for categorical data. Statistical significance was defined as a two-sided P-value below 0.05.

Before logistic regression, statistical assumptions were checked. Multicollinearity was assessed using variance inflation factors (VIF); all VIF values were below the conventional threshold of 5 (range: 1.01–1.47), indicating no substantial multicollinearity among the independent variables (**Supplementary Figure S1**).

To evaluate the association between each adiposity parameter and elevated blood pressure, we built three nested logistic regression frameworks incorporating progressively broader covariate adjustment. Model 1 was unadjusted (crude analysis). Model 2 was adjusted for chronological age and biological sex. Model 3 was adjusted for age, sex, diabetes duration, HbA1c, eGFR, smoking status, alcohol consumption, and antidiabetic medication use.

CVAI and LAP were incorporated both as continuous parameters (per 0.1-unit increment) and as ordinal categories (quartiles, utilizing Q1 as the reference) according to their distribution across the entire study cohort. A trend P-value was derived by inputting the midpoint of each quartile as a continuous term in the logistic regression equation. Model 3 functioned as the principal analytical framework for drawing inferences.

To characterize potential curvilinear associations linking adiposity metrics to hypertension, restricted cubic spline (RCS) regression incorporating 4 knots positioned at the 5th, 35th, 65th, and 95th percentiles of the exposure distribution was employed. The reference value corresponded to the median of each parameter. The global association (P for overall) and the deviation from linearity (P for non-linearity) were quantified via likelihood ratio tests. The horizontal axis was truncated at the 99.5th percentile for LAP and the 99.8th percentile for CVAI to eliminate extreme values and enhance graphical clarity. A segmented linear regression framework was additionally applied to detect potential threshold effects of LAP on hypertensive outcomes. The inflection point was estimated employing a recursive algorithm, and the likelihood ratio test was utilized to contrast the segmented model against the conventional linear model.

Stratified analyses by gender were executed to determine whether sex moderates these associations. Subgroup evaluations were carried out stratified according to gender and metformin utilization to gauge the stability of observations. Interaction P-values were computed employing likelihood ratio tests comparing frameworks with and without the interaction product between the adiposity parameter and the stratification variable. Subgroup findings were depicted using forest plot diagrams.

The principal analytical approach employed multiple imputation through chained equations (MICE) for managing absent covariate information. This was implemented using the Fully Conditional Specification (FCS) framework (21). Each parameter received its own imputation algorithm. Predefined models accommodated continuous data (predictive mean matching, normal distribution), binary data (logistic regression), nominal categorical data (polytomous logistic regression), and ordinal categorical data (proportional odds model). To ensure variable-level coherence, passive imputation techniques were incorporated. Twenty complete imputed datasets were produced, and pooled estimates were derived through Rubin’s combinatory rules. Graphical diagnostic outputs were created to evaluate imputation integrity. A sensitivity analysis was conducted using the complete case approach, in which participants with any missing values in the covariates were excluded (n = 3,537). Analysis was performed using R 4.2.2 (https://www.r-project.org/; The R Foundation, Vienna, Austria) and the Free Statistics software (version 2.3; Beijing FreeClinical Medical Technology Co., Ltd, Beijing, China).

## 3 Results

### 3.1 Baseline characteristics

Of the 4,155 participants with T2DM included in this study, 2,846 (68.5%) had hypertension and 1,309 (31.5%) were normotensive (**Table 1**). The mean age was 63.2 ± 11.7 years, with 2157 males (51.9%) and 1998 females (48.1%). The median diabetes duration was 8.0 (IQR: 2.0–12.0) years. The mean HbA1c was 8.4 ± 2.1%, and the mean eGFR was 103.5 ± 41.8 mL/min/1.73 m².

**Table 1:** Baseline characteristics of type 2 diabetes mellitus patients stratified by hypertension status.

Characteristics	Total (n = 4155)	Normotensive (n = 1309)	Hypertension (n = 2846)	P-value
Demographic characteristics				
Sex, n (%)				0.302
Male	2157 (51.9)	695 (53.1)	1462 (51.4)	
Female	1998 (48.1)	614 (46.9)	1384 (48.6)	
Age, years	63.2 ± 11.7	60.2 ± 12.6	64.6 ± 11.0	< 0.001
Lifestyle factors				
Smoking history, n (%)				0.021
No	3652 (87.9)	1128 (86.2)	2524 (88.7)	
Yes	503 (12.1)	181 (13.8)	322 (11.3)	
Drinking history, n (%)				0.79
No	3764 (91.0)	1182 (90.9)	2582 (91.1)	
Yes	371 (9.0)	119 (9.1)	252 (8.9)	
Diabetes-related characteristics				
Duration of diabetes, years	8.0 (2.0, 12.0)	7.0 (2.0, 11.0)	8.0 (3.0, 14.0)	0.002
HbA1c, %	8.4 ± 2.1	8.7 ± 2.3	8.3 ± 2.1	< 0.001
Laboratory parameters				
eGFR, mL/min/1.73 m²	103.5 ± 41.8	114.8 ± 43.0	98.3 ± 40.2	< 0.001
Triglycerides, mmol/L	1.5 (1.0, 2.2)	1.4 (0.9, 2.1)	1.5 (1.0, 2.2)	0.006
Total cholesterol, mmol/L	4.4 ± 1.4	4.6 ± 1.5	4.4 ± 1.4	< 0.001
HDL-C, mmol/L	1.2 ± 0.4	1.2 ± 0.5	1.2 ± 0.4	0.024
LDL-C, mmol/L	2.6 (1.9, 3.2)	2.7 (2.0, 3.3)	2.5 (1.8, 3.2)	< 0.001
Antidiabetic medications, n (%)				
SGLT2 inhibitors	760 (18.3)	230 (17.6)	530 (18.6)	0.412
α-glycosidase inhibitor	1148 (27.6)	371 (28.4)	777 (27.3)	0.477
SU	469 (11.3)	132 (10.1)	337 (11.8)	0.096
Metformin	1604 (38.6)	490 (37.4)	1114 (39.1)	0.293
Insulin use	1519 (36.6)	503 (38.5)	1016 (35.7)	0.089
DPP-4 inhibitors	266 (6.4)	81 (6.2)	185 (6.5)	0.7
GLP-1 receptor agonists	55 (1.3)	20 (1.5)	35 (1.2)	0.435
TZD	91 (2.2)	39 (3.0)	52 (1.8)	0.018
Repaglinide	152 (3.7)	50 (3.8)	102 (3.6)	0.707
Anthropometric and obesity indices				
BMI, kg/m²	26.3 ± 8.2	25.5 ± 9.9	26.6 ± 7.2	< 0.001
Waist circumference, cm	83.3 ± 11.9	81.3 ± 11.8	84.2 ± 11.9	< 0.001
CVAI	111.1 ± 46.7	98.5 ± 49.0	116.8 ± 44.4	< 0.001
LAP	30.1 (16.4, 52.8)	26.5 (13.1, 47.9)	31.7 (18.3, 55.4)	< 0.001

BMI, body mass index; CHOL, total cholesterol; CVAI, Chinese visceral adiposity index; DPP-4, dipeptidyl peptidase-4; eGFR, estimated glomerular filtration rate; GLP-1, glucagon-like peptide-1; HbA1c, glycated hemoglobin; HDL-C, high-density lipoprotein cholesterol; IQR, interquartile range; LAP, lipid accumulation product; LDL-C, low-density lipoprotein cholesterol; SGLT2, sodium-glucose cotransporter-2; SD, standard deviation; SU, sulfonylureas; TG, triglycerides; TZD, thiazolidinediones.

Data are presented as mean ± standard deviation for normally distributed continuous variables, median (interquartile range) for skewed continuous variables, and number (percentage) for categorical variables. The p-values were calculated using the independent samples t-test or Mann-Whitney U test for continuous variables, and the chi-square test or Fisher’s exact test for categorical variables, as appropriate.

Compared with normotensive participants, those with hypertension were older (64.6 ± 11.0 vs. 60.2 ± 12.6 years; P < 0.001), had longer diabetes duration [8.0 (3.0–14.0) vs. 7.0 (2.0–11.0) years; P = 0.002], lower HbA1c (8.3 ± 2.1% vs. 8.7 ± 2.3%; P < 0.001), lower eGFR (98.3 ± 40.2 vs. 114.8 ± 43.0 mL/min/1.73 m²; P < 0.001), and higher BMI (26.6 ± 7.2 vs. 25.5 ± 9.9 kg/m²; P < 0.001). The prevalence of smoking history was lower in hypertensive participants (11.3% vs. 13.8%; P = 0.021). No significant differences were observed in sex, drinking history, or use of most antidiabetic medications between groups (all P > 0.05). CVAI (116.8 ± 44.4 vs. 98.5 ± 49.0; P < 0.001) and LAP [31.7 (18.3–55.4) vs. 26.5 (13.1–47.9); P < 0.001] were higher in hypertensive participants (**Table 1**).

### 3.2 Association between CVAI and hypertension

In the fully adjusted model (Model 3), both CVAI and LAP were positively associated with hypertension (**Table 2**). CVAI showed a clear dose–response relationship with hypertension. Specifically, for every 0.1-unit increase in CVAI, the odds of hypertension increased by 11% (OR = 1.11, 95% CI: 1.09–1.13; P < 0.001). Consistently, compared with participants in Q1, those in Q2, Q3, and Q4 had progressively higher odds of hypertension, with ORs of 1.59 (95% CI: 1.32–1.92), 2.45 (95% CI: 2.00–2.99), and 3.27 (95% CI: 2.66–4.02), respectively (P for trend < 0.001).

**Table 2:** Association between obesity indices and hypertension in patients with type 2 diabetes mellitus (multiple imputation).

Variable	n	Events, n (%)	Model 1		Model 2		Model 3	
			OR (95% CI)	*P*-value	OR (95% CI)	*P*-value	OR (95% CI)	*P*-value
CVAI								
Per 0.1-unit increase	4155	2846 (68.5)	1.11 (1.09–1.13)	<0.001	1.11 (1.09–1.13)	<0.001	1.11 (1.09–1.13)	<0.001
Quartiles								
Q1	1039	567 (54.6)	1 (Ref)	—	1 (Ref)	—	1 (Ref)	—
Q2	1038	675 (65.0)	1.55 (1.30–1.85)	<0.001	1.56 (1.30–1.87)	<0.001	1.59 (1.32–1.92)	<0.001
Q3	1039	777 (74.8)	2.47 (2.05–2.97)	<0.001	2.44 (2.01–2.97)	<0.001	2.45 (2.00–2.99)	<0.001
Q4	1039	827 (79.6)	3.25 (2.67–3.94)	<0.001	3.28 (2.69–4.01)	<0.001	3.27 (2.66–4.02)	<0.001
*P* for trend				<0.001		<0.001		<0.001
LAP								
Per 0.1-unit increase	4155	2846 (68.5)	1.01 (1.00–1.02)	0.195	1.03 (1.02–1.04)	<0.001	1.03 (1.02–1.05)	<0.001
Quartiles								
Q1	1038	620 (59.7)	1 (Ref)	—	1 (Ref)	—	1 (Ref)	—
Q2	1039	723 (69.6)	1.54 (1.29–1.85)	<0.001	1.73 (1.43–2.08)	<0.001	1.70 (1.41–2.06)	<0.001
Q3	1038	743 (71.6)	1.70 (1.41–2.04)	<0.001	2.09 (1.72–2.53)	<0.001	2.12 (1.74–2.57)	<0.001
Q4	1040	760 (73.1)	1.83 (1.52–2.20)	<0.001	2.90 (2.37–3.56)	<0.001	3.01 (2.43–3.72)	<0.001
*P* for trend				<0.001		<0.001		<0.001

CI, confidence interval; CVAI, Chinese visceral adiposity index; LAP, lipid accumulation product; OR, odds ratio; Q, quartile; Ref, reference.

Model 1: unadjusted logistic regression. Model 2 is adjusted for age, sex. Model 3 is adjusted for age, sex, diabetes duration, glycated hemoglobin, estimated glomerular filtration rate, smoking status, alcohol consumption, and use of antidiabetic medications. Quartiles were categorized based on the distribution of each obesity index in the total study cohort (Q1: lowest; Q4: highest). *P* for trend was calculated by entering the median value of each quartile as a continuous variable in the logistic regression model.

A sex-stratified RCS model was used to assess the dose–response association between CVAI and the predicted probability of hypertension separately in males and females (**Figure 2**). Overall, the probability of hypertension increased with higher CVAI in both groups. The spline suggested an approximately linear trend in males (P for non-linearity = 0.177), whereas the evidence for nonlinearity in females was weaker and approached borderline significance (P for non-linearity = 0.055).

**Figure 2:**
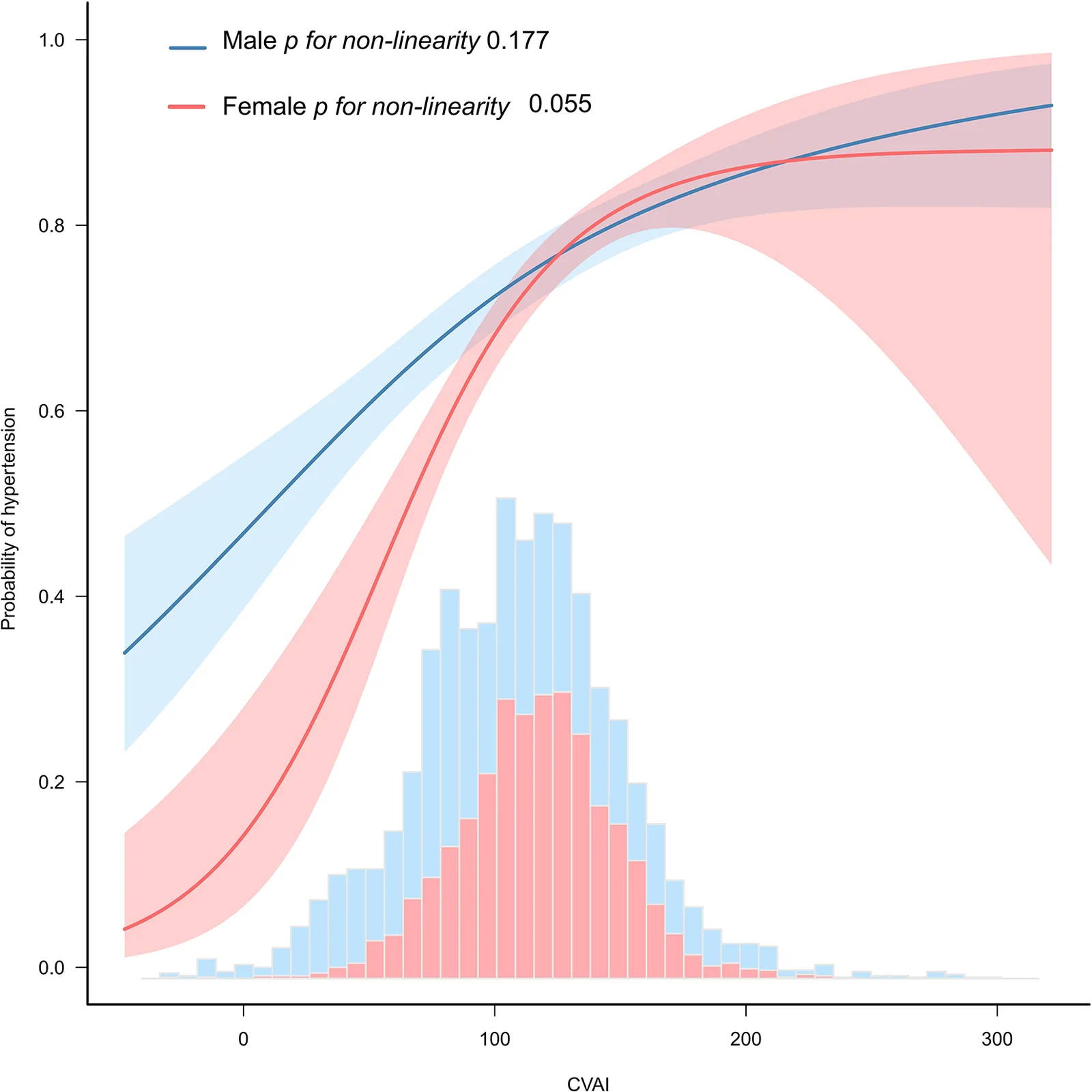
Sex-specific restricted cubic spline analysis of the association between Chinese visceral adiposity index (CVAI) and the predicted probability of hypertension in patients with type 2 diabetes mellitus. The solid blue and red lines represent the adjusted predicted probabilities of hypertension for males and females, respectively. The shaded areas indicate the corresponding 95% confidence intervals. The histograms at the bottom display the frequency distributions of CVAI in males (blue) and females (red). The x-axis was truncated at the 99.8th percentile of the CVAI distribution to exclude extreme outliers and improve visualization. All models were adjusted for age, diabetes duration, glycated hemoglobin, estimated glomerular filtration rate, smoking status, alcohol consumption, and use of antidiabetic medications. *P* for non-linearity = 0.177 in males and *P* for non-linearity = 0.055 in females. CI, confidence interval; CVAI, Chinese visceral adiposity index; RCS, restricted cubic spline.

### 3.3 Association between LAP and hypertension

LAP was positively associated with hypertension. Specifically, for every 0.1-unit increase in LAP, the odds of hypertension increased by 3% (OR = 1.03, 95% CI: 1.02–1.05; P < 0.001). Consistently, compared with participants in Q1, those in Q2–Q4 had progressively higher odds of hypertension, with ORs of 1.70 (95% CI: 1.41–2.06), 2.12 (95% CI: 1.74–2.57), and 3.01 (95% CI: 2.43–3.72), respectively (P for trend < 0.001) (**Table 2**).

**Figure 3** shows the RCS analysis of the association between LAP and hypertension. The model demonstrated a significant overall positive association (P for overall < 0.001) and a significant deviation from linearity (P for non-linearity < 0.001), indicating that the relationship between LAP and hypertension is not purely linear. Specifically, the adjusted OR for hypertension increased as LAP rose at lower levels, and the increase leveled off (plateaued) at higher LAP levels.

**Figure 3:**
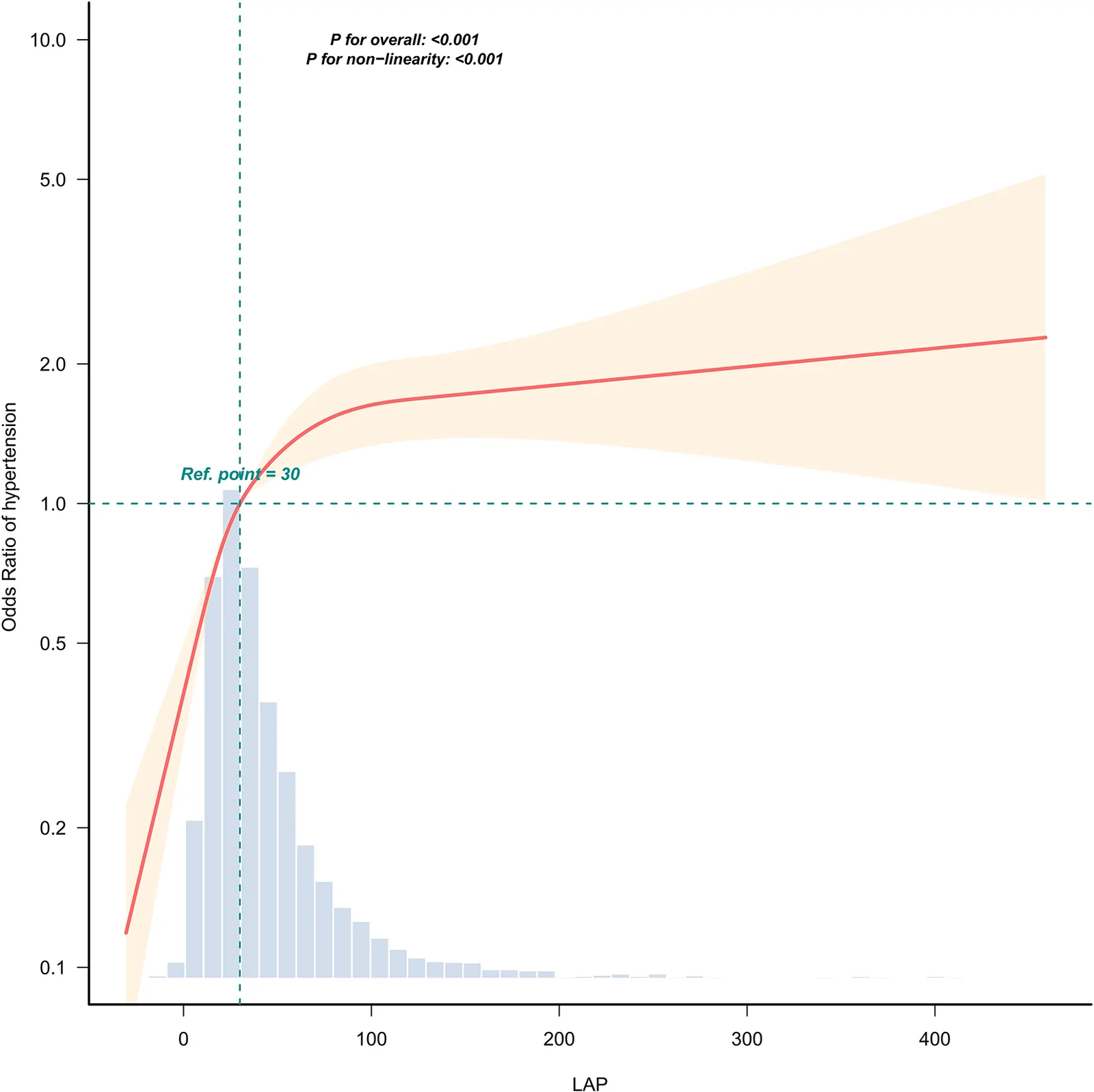
Restricted cubic spline analysis of the association between lipid accumulation product (LAP) and the odds of hypertension in patients with type 2 diabetes mellitus. The solid red line represents the adjusted odds ratio (OR), and the shaded orange area indicates the 95% confidence interval (CI). The reference point was set at LAP = 30 (indicated by the vertical dashed line). The horizontal dashed line denotes an OR of 1.0. The histogram at the bottom displays the frequency distribution of LAP in the study population. The x-axis was truncated at the 99.5th percentile of the LAP distribution to exclude extreme outliers and improve visualization. The model was adjusted for age, sex, diabetes duration, glycated hemoglobin, estimated glomerular filtration rate, smoking status, alcohol consumption, and use of antidiabetic medications. *P* for overall association <0.001; *P* for non-linearity <0.001. CI, confidence interval; LAP, lipid accumulation product; OR, odds ratio; RCS, restricted cubic spline.

The presence of a threshold relationship between LAP and hypertension was examined with a two-piecewise linear regression model, and a turning point was found at LAP = 44.187 (95% CI: 42.141–46.233) (**Table 3**; **Figure 3**). For LAP values less than this cutoff, higher LAP corresponded to greater odds of hypertension; namely, each 0.1 unit rise in LAP was linked to an OR of 1.028 (95% CI: 1.021–1.035; P < 0.001). Conversely, once LAP surpassed the threshold, the LAP–hypertension association became negligible and failed to achieve statistical significance (OR = 1.001, 95% CI: 0.999–1.004; P = 0.185).

**Table 3:** Threshold effect analysis of the association between LAP and hypertension in patients with type 2 diabetes mellitus.

Parameter	n	Estimate (95% CI)	*P*-value
Breakpoint	4134	44.187 (42.141–46.233)	—
Effect below breakpoint	2819	1.028 (1.021–1.035)	<0.001
Effect above breakpoint	1315	1.001 (0.999–1.004)	0.185
Likelihood ratio test	—	—	<0.001

CI, confidence interval; LAP, lipid accumulation product.

Threshold effect analysis was performed using a two-piecewise linear regression model. The adjusted model was adjusted for age, sex, diabetes duration, glycated hemoglobin, estimated glomerular filtration rate, smoking status, alcohol consumption, and use of antidiabetic medications. The breakpoint represents the estimated threshold of LAP at which the association with hypertension changes. Effect sizes below and above the breakpoint represent the odds ratios per 0.1 unit increase in LAP within each segment. The likelihood ratio test was used to compare the threshold effect model with the standard linear model.

### 3.4 Subgroup analyses

Subgroup analyses were carried out after stratifying participants by sex and metformin use (**Figure 4**). Forest plots in **Figure 4** present the adjusted ORs with 95% CIs for hypertension across quartiles of CVAI (**Figure 4A**) and LAP (**Figure 4B**), enabling comparison of the association within different subgroups.

**Figure 4:**
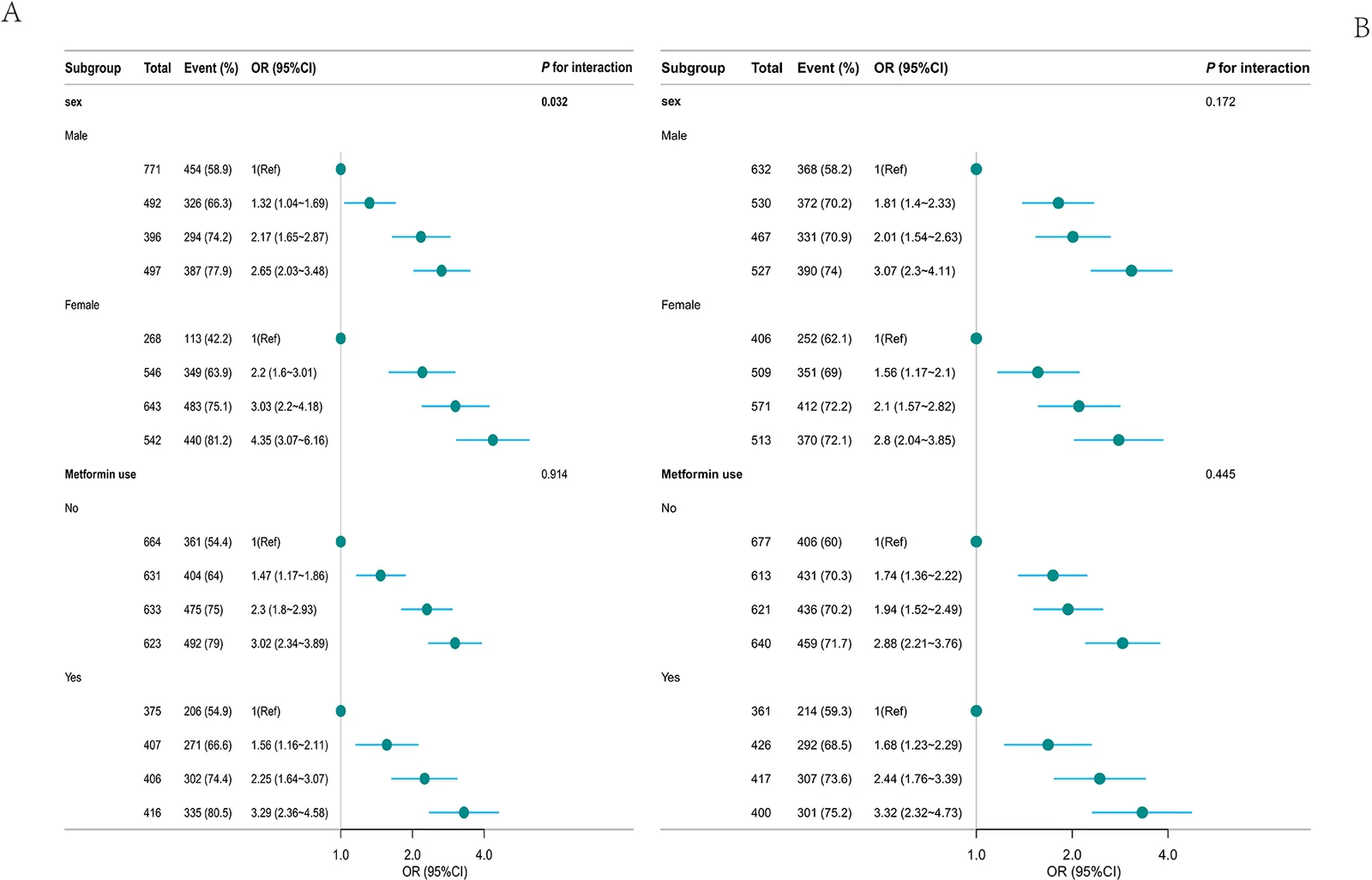
Subgroup analyses of the association between obesity indices and hypertension in patients with type 2 diabetes mellitus. Forest plots display the adjusted odds ratios (ORs) and 95% confidence intervals (CIs) for hypertension across quartiles of **(A)** Chinese visceral adiposity index (CVAI) and **(B)** lipid accumulation product (LAP), stratified by sex and metformin use. The vertical dashed line represents an OR of 1.0. All models were adjusted for age, sex (when not stratified), diabetes duration, glycated hemoglobin, estimated glomerular filtration rate, smoking status, alcohol consumption, and use of antidiabetic medications (except metformin when stratified by metformin use). *P* for interaction was calculated using likelihood ratio tests comparing models with and without the interaction term between the obesity index and the stratification variable. CI, confidence interval; CVAI, Chinese visceral adiposity index; LAP, lipid accumulation product; OR, odds ratio; Ref, reference.

Stratified results indicated that greater CVAI and LAP corresponded to higher odds of hypertension across all examined strata. With respect to CVAI, the Q4 versus Q1 ORs fell between 2.86 and 3.86. Relative to Q1, Q4 was associated with an OR of 3.86 (95% CI: 2.82–5.28) in men and 2.86 (95% CI: 2.06–3.96) in women. In addition, among individuals receiving metformin, the Q4 OR reached 3.44, whereas it was 3.14 for those not using metformin.

Likewise, for LAP, positive associations were present in each subgroup, and the Q4 versus Q1 ORs ranged from 2.82 to 3.52. Specifically, the Q4 OR was 3.52 (95% CI: 2.44–5.08) for females and 2.82 (95% CI: 2.10–3.80) for males. Notably, no meaningful effect modification was detected; interaction P values for all stratification variables were >0.05, suggesting that the associations were generally comparable across strata.

### 3.5 Sensitivity analysis

To test whether our key conclusions were reliable, we performed a sensitivity analysis restricted to participants with complete data (n = 3,537) (**Supplementary Table S1**). After full adjustment, higher CVAI continued to be linked with hypertension. In particular, a 0.1-unit elevation in CVAI was related to hypertension with an OR of 1.11 (95% CI: 1.09–1.13; P < 0.001), and the odds rose across CVAI quartiles (Q2: OR = 1.56 (95% CI: 1.28–1.92); Q3: OR = 2.44 (95% CI: 1.96–3.03); Q4: OR = 3.36 (95% CI: 2.69–4.21). A comparable trend was seen for LAP: each 0.1-unit increase in LAP corresponded to an OR of 1.03 (95% CI: 1.02–1.04; P < 0.001), with quartile-specific ORs of 1.69 (95% CI: 1.37–2.08), 2.21 (95% CI: 1.78–2.75), and 3.13 (95% CI: 2.48–3.94) for Q2, Q3, and Q4, respectively (P for trend < 0.001).

## 4 Discussion

This cross-sectional investigation evaluated two visceral adiposity measures, namely the Chinese visceral adiposity index (CVAI) and the lipid accumulation product (LAP), in terms of their associations with hypertension among a large cohort of 4,155 Chinese patients with type 2 diabetes mellitus (T2DM), incorporating sex-stratified analyses. The principal observations are summarized below. First, following full covariate adjustment, both CVAI and LAP demonstrated independent associations with hypertension, yet their dose-response profiles differed: CVAI displayed a sustained linear pattern, whereas LAP exhibited a nonlinear relationship characterized by a saturation effect. Second, although both indices showed robust associations, CVAI consistently yielded numerically higher odds ratios across quartiles compared with LAP in this sample. Third, sex did not substantially modify these associations, as interaction P-values for both indices were non-significant; however, sex-stratified spline analyses hinted at potential divergence, with CVAI showing an approximately linear trend in males but borderline non-linearity in females.

Ectopic fat accumulation and lipotoxicity are recognized as key risk factors for hypertension, particularly through mechanisms involving renin-angiotensin-aldosterone system activation and sympathetic overdrive (22, 23). In the present investigation, both CVAI and LAP showed positive associations with hypertensive status, a pattern aligning with earlier population-level evidence from China (4, 19). By contrast, a community-based study in a general adult population found a weaker association between LAP and blood pressure, potentially due to the inclusion of younger, healthier individuals (24). Several elements may underlie this divergence. The earlier investigation enrolled participants spanning from 18 years of age, whereas our cohort comprised exclusively T2DM patients whose average age exceeded 60 years. Visceral adipose tissue may exert more pronounced metabolic consequences in older individuals with diabetes, reflecting age-associated sarcopenia and adipose redistribution that amplify insulin resistance and endothelial impairment (25). Moreover, at any given LAP value, Chinese adults typically harbor a larger percentage of body fat and more extensive visceral adiposity than Western individuals (26, 27), which could render lipid-based composite markers such as LAP more responsive within Asian populations (28). Variation in covariate handling, particularly adjustment for glucose-lowering agents capable of altering body weight and lipid parameters, might likewise play a role (29). The CVAI-hypertension relationship observed here parallels findings reported in additional Chinese cohorts (4, 14). Notably, data from a national investigation indicated that the incremental hypertension risk per unit rise in CVAI was larger in Chinese populations, particularly among those with pre-existing metabolic conditions, relative to Western populations (10). CVAI was originally introduced in 2016 to estimate visceral fat area through sex-specific formulas validated against magnetic resonance imaging (15). Furthermore, a recent longitudinal study tracking Chinese adults over five years delineated three distinct CVAI trajectories, and participants in the “elevated-increasing” cluster faced markedly elevated hypertension risks, highlighting the inherently dynamic character of this association (17).

A distinct relationship profile emerged for each adiposity measure with respect to hypertension. In both genders, CVAI displayed a steady linear positive trend spanning its full range, while LAP exhibited a marked threshold phenomenon, with its link to hypertension leveling off once the index exceeded roughly 44.2. This divergence could point to differential biological responsiveness along the obesity spectrum; however, given the cross-sectional design of this investigation, definitive statements regarding causality are not possible. The plateauing effect of LAP suggests that once central lipid overaccumulation reaches a critical threshold, additional increases may not proportionally elevate hypertension risk, possibly due to ceiling effects in vascular remodeling or the saturation of lipid-induced inflammatory pathways (11, 23). The more pronounced associations at lower LAP levels imply that these indices may be particularly sensitive to early metabolic deteriorations, aligning with the “metabolically obese normal-weight” phenotype, where normal BMI belies underlying visceral adiposity (26).

The divergent dose–response profiles can be understood in light of the compositional differences between the two indices. CVAI incorporates age and HDL-cholesterol in addition to anthropometric and triglyceride components, and is calibrated against imaging-measured visceral fat; it therefore rises monotonically with the cumulative, age-related metabolic exposure that drives hypertension, yielding a linear association across its full range. LAP, by contrast, is a simple waist–triglyceride product that reflects systemic lipid overaccumulation without adjustment for age or high-density lipoprotein; its association with hypertension accordingly plateaus once lipid accumulation exceeds a critical threshold, suggesting a saturation of the pathways through which ectopic lipid promotes blood-pressure elevation. The consistently higher odds ratios for CVAI across quartiles further indicate that visceral adiposity—rather than systemic lipid overaccumulation alone—is the more relevant driver of hypertension in this population. These differences do not imply that one index is uniformly superior; rather, CVAI and LAP interrogate related but distinct dimensions of adiposity-related risk.

Sex-stratified analyses revealed broadly consistent associations, with interaction P-values >0.05, suggesting that the overall relationship between these adiposity indices and hypertension is comparable between males and females in this T2DM cohort. However, subtle differences in the dose-response shape merit mechanistic consideration. In males, CVAI showed a strictly linear relationship, whereas in females, the non-linear trend approached significance. Several mechanisms may help interpret these sex-specific contrasts. In men, androgens encourage the preferential storage of fat within the abdominal cavity (30), and the TG and HDL-C components contained in CVAI faithfully mirror the lipid derangements generated by this visceral fat accumulation, thus producing a smooth linear risk slope. Conversely, estrogen withdrawal after menopause in women raises circulating triglycerides and drives adipose tissue toward central compartments, which may make WC-TG-based markers such as LAP particularly informative at modest values, although at higher values the association tends to flatten because generalized obesity exerts a masking effect (12, 31–33). Given that every participant in this cohort was at least 18 years old (mean age, 63 years), transferring these results to younger diabetic patients or to individuals without diabetes merits careful judgment.

Although all models were adjusted for nine antidiabetic drug classes and metformin-stratified subgroup analyses yielded consistent results, pharmacotherapy effects warrant consideration. Agents promoting weight loss and lipid improvement — notably GLP-1 receptor agonists and SGLT2 inhibitors (34, 35) — would attenuate rather than inflate the observed associations. Because hypertensive status was partly defined by antihypertensive treatment, well-controlled patients remained classified as hypertensive; this non-differential misclassification would bias results toward the null. Residual confounding by unmeasured psychosocial factors (36, 37) cannot be excluded.

The present study is subject to several limitations. First, the cross-sectional design prevents causal inference; reverse causality—whereby hypertension-related hemodynamic changes might influence lipid metabolism and central adiposity—cannot be ruled out. Second, although CVAI and LAP are validated research surrogates, they are not yet integrated into routine clinical guidelines, limiting their immediate translational utility. Third, the single-center, retrospective design based on electronic medical records may introduce selection bias, and the relatively high proportion of excluded patients due to missing LAP/CVAI data (n = 1,515) might affect generalizability. Fourth, we lacked data on menopausal status, antihypertensive medication classes, dietary factors, and psychosocial variables (e.g., chronic stress and emotional distress), which could confound or modify the observed relationships. Despite these limitations, this study has notable strengths, including a large T2DM-specific sample, rigorous multiple imputation for missing covariates, comprehensive adjustment for antidiabetic regimens, and the use of both RCS and threshold effect analyses to delineate non-linear patterns, thereby providing a more nuanced understanding of adiposity–hypertension links in this high-risk population.

## 5 Conclusion

This cross-sectional study of 4,155 Chinese type 2 diabetes patients compared two visceral adiposity indices—CVAI and LAP—for hypertension associations. Both were independently associated with hypertension, but dose-response patterns differed: CVAI showed a linear risk gradient, while LAP exhibited non-linearity with a saturation plateau near 44.2. CVAI had slightly higher odds ratios (Q4 OR: 3.27 vs. 3.01). Sex did not significantly modify associations (all P for interaction > 0.05), though spline analyses suggested CVAI trends may approach non-linearity in females. As a single-center cross-sectional study, causality cannot be established, but findings generate the hypothesis that visceral adiposity indices—particularly LAP—may have biological ceilings in T2DM-related hypertension, warranting prospective validation in larger multicenter cohorts.

## Data Availability

The raw data supporting the conclusions of this article will be made available by the authors, without undue reservation.

## Supplementary material

Supplementary Figure 1 Collinearity diagnostics of the multivariable logistic regression model. Bar plot displaying the variance inflation factor (VIF) for each covariate included in the adjusted logistic regression model. The horizontal dashed red line indicates the conventional threshold of VIF = 5. All variables showed VIF values below this threshold (range: 1.01–1.47), indicating no substantial multicollinearity among the independent variables. CVAI, Chinese visceral adiposity index; DPP-4, dipeptidyl peptidase-4 inhibitor; eGFR, estimated glomerular filtration rate; GLP-1, glucagon-like peptide-1 receptor agonist; HbA1c, glycated hemoglobin; LAP, lipid accumulation product; SGLT2, sodium-glucose cotransporter-2 inhibitor; SU, sulfonylurea; TZD, thiazolidinedione; VIF, variance inflation factor.
