# Supplementary material for: The association between obesity index and hypertension in type 2 diabetes: a cross-sectional study
Source: Front Endocrinol (Lausanne). 2026 Oct 27;17:1998153. doi: 10.3389/fendo.2026.1998153 (PMC13747799; doi:10.3389/fendo.2026.1998153)
Supplement: Supplementary file 1 [file Table1.docx]

****Table S1. Association between obesity indices and hypertension in patients with type 2 diabetes mellitus (complete case analysis).****

| Variable | n | Events, n (%) | Model 1 | | Model 2 | |
| --- | --- | --- | --- | --- | --- | --- |
|  |  |  | OR (95% CI) | *P*-value | OR (95% CI) | *P*-value |
| CVAI |  |  |  |  |  | |
| Per 0.1-unit increase | 3537 | 2438 (68.9) | 1.11 (1.09–1.13) | <0.001 | 1.11 (1.09–1.13) | <0.001 |
| Quartiles |  |  |  |  |  |  |
| Q1 | 875 | 484 (55.3) | 1 (Ref) | — | 1 (Ref) | — |
| Q2 | 896 | 583 (65.1) | 1.50 (1.24–1.82) | <0.001 | 1.56 (1.28–1.92) | <0.001 |
| Q3 | 873 | 654 (74.9) | 2.41 (1.97–2.96) | <0.001 | 2.44 (1.96–3.03) | <0.001 |
| Q4 | 893 | 717 (80.3) | 3.29 (2.66–4.07) | <0.001 | 3.36 (2.69–4.21) | <0.001 |
| *P* for trend |  |  |  | <0.001 |  | <0.001 |
| LAP |  |  |  |  |  |  |
| Per 0.1-unit increase | 3537 | 2438 (68.9) | 1.01 (1.00–1.02) | 0.187 | 1.03 (1.02–1.04) | <0.001 |
| Quartiles |  |  |  |  |  |  |
| Q1 | 856 | 510 (59.6) | 1 (Ref) | — | 1 (Ref) | — |
| Q2 | 896 | 623 (69.5) | 1.55 (1.27–1.89) | <0.001 | 1.69 (1.37–2.08) | <0.001 |
| Q3 | 877 | 632 (72.1) | 1.75 (1.43–2.14) | <0.001 | 2.21 (1.78–2.75) | <0.001 |
| Q4 | 908 | 673 (74.1) | 1.94 (1.59–2.38) | <0.001 | 3.13 (2.48–3.94) | <0.001 |
| *P* for trend |  |  |  | <0.001 |  | <0.001 |

****Abbreviations:**** CI, confidence interval; CVAI, Chinese visceral adiposity index; LAP, lipid accumulation product; OR, odds ratio; Q, quartile; Ref, reference.

****Notes:** This sensitivity analysis was conducted using the complete case approach, in which participants with any missing values in the covariates were excluded (n = 3,537). The main analysis was performed using multiple imputation by chained equations (MICE) to account for missing data. Model 1 is unadjusted. Model 2: multivariable logistic regression adjusted for age, sex, diabetes duration, glycated hemoglobin, estimated glomerular filtration rate, smoking status, alcohol consumption, and use of antidiabetic medications. CVAI and LAP quartiles were categorized based on the distribution in the complete case cohort (Q1: lowest; Q4: highest). P for trend was calculated by entering the median value of each quartile as a continuous variable in the logistic regression model.**
